# Health professionals’ and researchers’ opinions on conducting clinical deprescribing trials

**DOI:** 10.1002/prp2.476

**Published:** 2019-04-25

**Authors:** Alexander J. Clough, Sarah N. Hilmer, Lisa Kouladjian‐O'Donnell, Sharon L. Naismith, Danijela Gnjidic

**Affiliations:** ^1^ School of Pharmacy University of Sydney Camperdown NSW Australia; ^2^ Kolling Institute of Medical Research University of Sydney and Royal North Shore Hospital St Leonards NSW Australia; ^3^ Brain & Mind Centre University of Sydney Camperdown NSW Australia; ^4^ Charles Perkins Centre University of Sydney Camperdown NSW Australia

**Keywords:** clinical trials, methodology, prescribing, quality use of medicines

## Abstract

While clinical deprescribing trials are increasingly being performed, there is no guidance on the optimum conduction of such studies. The aim of this survey was to explore the perspectives, attitudes, interests, barriers, and enablers of conducting clinical deprescribing trials among health professionals and researchers. An anonymous survey was developed, reviewed, and piloted by all investigators and informed by consultation with experts, as well as current deprescribing guidelines. The questions were formulated around current clinical trial frameworks and incorporated identified enablers and barriers of performing deprescribing studies. The survey was sent to members of Australian and international deprescribing, pharmacological, and pharmacy organizations, and other researchers published in deprescribing. A total of 96 respondents completed the survey (92.3% completion rate). Respondents indicated the main deprescribing trial rationale is to generate evidence to optimize patient‐centered outcomes (79.2%). Common barriers identified included the time and effort required (18.2%), and apprehension of health professionals involved in trials (17.1%). Studies are enabled by positive attitudes toward deprescribing of treating prescribers (24.4%) and patients (20.9%). Classical randomized controlled trials (RCTs) were deemed the most appropriate methodology (93.2%). Sixty percent of participants indicated a good clinical practice framework is required to guide the conduct of deprescribing trials. There were no significant differences in responses based on previous experience in conducting clinical deprescribing trials. In conclusion, clinical deprescribing trials should be conducted to investigate whether deprescribing medications improves patient care. A future deprescribing trial framework should use classical RCTs as a model, ensure participant safety, and target patient‐centered outcomes.

**Abbreviations**
ADeN: Australian Deprescribing Network
APSA: Australasian Pharmaceutical Science Association
ASCEPT: Australasian Society of Clinical and Experimental Pharmacologists and Toxicologists
CHERRIES: Checklist for Reporting Results of Internet E‐Surveys
IGRIMUP: Inappropriate Medication Use and Polypharmacy Group
MeSH: Medical Subject Heading
PPI: proton‐pump inhibitor
RCT: randomized controlled trials
REDCap: Research Data Electronic Capture

## 1 INTRODUCTION

Deprescribing has been identified as the patient‐centered process of withdrawing potentially harmful or unnecessary medications in order to improve health outcomes.^1^ Despite many deprescribing studies being in progress internationally, there is an ongoing recognition of the need to conduct quality, robust clinical deprescribing trials to investigate the benefits and safety of stopping medicines.^2^, ^3^, ^4^ However, study design and outcomes often vary, leading to great heterogeneity in the literature, and presenting challenges for researchers and practitioners to synthesize results and implement recommendations into clinical practice.^5^, ^6^, ^7^, ^8^

The primary challenge in deprescribing research is the weighing of risks the patient may accept against the potential benefits of discontinuing a drug.^9^ The recognition of more information on appropriate medication use has been highlighted by the World Health Organization, who have established *Medication Without Harm* as the theme of the third Global Patient Safety Challenge, with an overall goal of reducing severe avoidable medication‐harm by 50% globally.^10^ To improve the knowledge on the safety of deprescribing, researchers and health professionals have called for more high‐quality evidence, requiring more clinical deprescribing trials.^11^, ^12^, ^13^ Definitive clinical deprescribing trials would not only inform on the safety of deprescribing but also provide guidance on assessment of relevant outcomes.

While there are international clinical deprescribing guidelines, there is no Consolidated Standards of Reporting Trials (CONSORT) extension nor recognized framework for conducting clinical deprescribing trials.^11^, ^13^, ^14^, ^15^, ^16^, ^17^ Furthermore, while numerous studies have examined the perspectives on deprescribing from health professionals and patient groups, only one has explored the perspectives of those individuals who conduct the deprescribing trials.^5^, ^12^, ^18^, ^19^, ^20^, ^21^, ^22^, ^23^, ^24^, ^25^, ^26^ However, this study only gathered the opinions of a selected group of researchers and health professionals in a research workshop setting, and did not systematically analyze the ideas brought forward to evaluate their recommendations.^12^, ^24^ Instead a World Café, open dialogue session with roundtable discussion, was used with three questions on: research priorities for developing; outcome measures to inform; and, how to evaluate the implementation of, deprescribing guidelines in clinical settings.^12^, ^24^ Nor did this study examine other themes specific to clinical trials such as participant recruitment, ethical approval barriers, or the most appropriate study design.

Given that increasing numbers of deprescribing trials are currently being conducted, direction is needed on their design, conduct, and reporting.^27^ Yet, at present, there is little data on health professionals’ and researchers’ perspectives and experiences about conducting deprescribing clinical trials.

## 2 AIM

To determine the perspectives, attitudes, interests, and perceived barriers and enablers in relation to conducting clinical deprescribing trials among health professionals and researchers.

## 3 MATERIALS AND METHODS

This cross‐sectional survey is reported per the Checklist for Reporting Results of Internet E‐Surveys (CHERRIES).^28^ Ethics approval for this study was granted by The University of Sydney's Human Research Ethics Committee, Sydney, Australia.

### 3.1 Design

An anonymous, online survey was created using Research Data Electronic Capture (REDCap) software hosted on University of Sydney servers, and consisted of a nonrandomized mix of multiple‐choice questions and open‐ended options.

Twelve questions were developed, reviewed and piloted by all investigators for content validity. In addition, we sought input on questionnaire content from key national (n = 2) and international (n = 2) experts with experience in conducting deprescribing trials. The questions were formulated based on current clinical trial frameworks and addressed themes identified as barriers and enablers in current literature.^15^ The final questionnaire consisted of 10 multiple‐choice questions (with open‐ended options) exploring participants’ experience and opinions on topics including trial rationale, and barriers and enablers across a clinical trial process. Finally, participants were asked if a clinical deprescribing trial framework needed to be developed, and whether the current CONSORT list [http://www.consort-statement.org/consort-statement/checklist ] required amending to include deprescribing trials. The final two questions included a free‐text component where participants could expand on their response. De‐identified basic sociodemographic data including age, gender, country of residence, and academic qualification were also captured.

The first page of the survey was the Participant Information Statement detailing the length of time of the survey, which data were stored and for how long, who the investigator was, and the purpose of the study. The final copy of the Letter of Invitation and the final survey is included in the Supplementary Information.

### 3.2 Participants

A letter of invitation with the closed survey link was sent by email to members of international deprescribing, and polypharmacy and pharmacy organizations including: Canadian Deprescribing Network (CaDeN); and Inappropriate Medication Use and Polypharmacy Group (IGRIMUP). National groups in Australia were also contacted and the letter of invitation and survey link distributed within monthly e‐newsletters (Australian Deprescribing Network [ADeN]; Australasian Society of Clinical and Experimental Pharmacologists and Toxicologists [ASCEPT]; Australasian Pharmaceutical Science Association [APSA]).

Further invitations were sent to any researchers not affiliated with the organizations above who had published deprescribing trials. These individuals were identified by searching the literature for studies under the Medical Subject Heading (MeSH) term “deprescribing” for publications that have deprescribing methodologies. The survey was open online for 10 weeks (June 23, 2017 to September 1, 2017) with two reminder emails also sent at approximately 3 weeks and 6 weeks after initial invitation. Cookies were used to assign a unique user identifier to each respondent who clicked the survey link and reached the Participant Information Statement on the first page of the survey.

### 3.3 Analysis of results

Anonymous data from the Research Electronic Data Capture (REDCap) website were imported into Microsoft Excel or Statistical Package for the Social Sciences (SPSS) Statistics for data analysis. Data were reported as descriptive statistics and presented as mean, number of responses, and percentage. The data from the open‐ended questions were derived using thematic analysis with themes derived using summative content analysis.^29^ Respondents were invited to identify three key components of a future framework, or to state three reasons why a future framework is not required. Study sample size was taken as the number of respondents who answered at least the first question following demographic data, irrespective of if all questions were answered or not.

## 4 RESULTS

**Figure 1:**
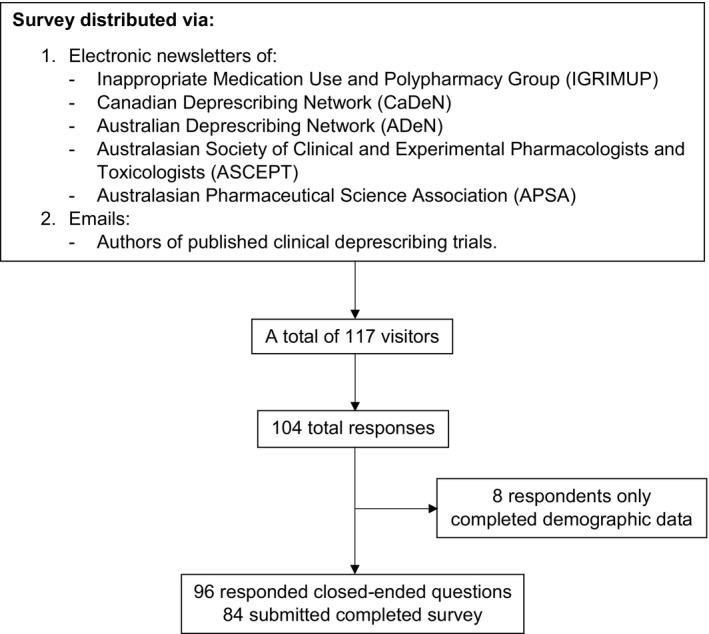
Respondent flowchart

Cookie data collected by REDCap indicated there were 117 unique site visitors with 104 respondents completing the baseline data (88.9% participation rate), and 96 completing at least one survey question (92.3% completion rate). Of these, 84 respondents submitted the completed survey with all questions answered (Figure 1).

The mean age of respondents was 45.0 (SD ± 11.6), and majority was female (54.3%) (Table 1). Canada was the most popular base for respondents (31.7%), and most respondents were academics (34.7%). Less than half (41.3%) of respondents indicated that they had previous experience in conducting clinical deprescribing trials. There were no significant differences between responses to all questions based on participants’ previous experience in conducting clinical deprescribing trials.

**Table 1:** Baseline demographic data of participants

Baseline demographics (N = 104)	
Age, mean (SD)	45.0 (±11.6)
Female, N (%)	57 (54.3)
Country, N (%)	
Canada	33 (31.7)
Australia	25 (24.0)
USA	12 (11.5)
Spain	6 (5.8)
UK	5 (4.8)
Ireland	4 (3.8)
Other	19 (18.3)
Profession, N (%)	
Academic	43 (34.7)
Pharmacist	37 (29.8)
Physician	28 (22.6)
Student Researcher	6 (4.8)
Nurse	5 (4.0)
Other	5 (4.0)
Experience in conducting deprescribing trials, N (%)	
Yes	43 (41.3)
No	61 (58.7)

### 4.1 Rationale, barriers, and enablers of conducting clinical deprescribing trials

In relation to survey sample size, 96 respondents completed at least one question and respondents could submit more than one response or skip questions giving different sample sizes for each question.

The first three questions explored the rationale, and common barriers and enablers of conducting clinical deprescribing trials (Table 2). Respondents overwhelmingly indicated that the primary rationale for deprescribing studies is to “optimise clinical and/or patient centered outcomes” (79.2 ± 8.1%). Common enablers to conducting trials were “the beliefs of other health professionals regarding benefits of deprescribing” (24.4%), and “willingness of patients to participate” (20.9%). Common barriers to completing trials were the “time and effort required” (18.2%), and “establishing and/or maintaining relationships with other health professionals” (17.1%).

**Table 2:** Responses to questions surveying the rationale, barriers, and enablers of conducting clinical deprescribing trials

Question theme	Number of responses (%)
Main rationale	96 (100)
Assess the efficacy of deprescribing interventions to optimize clinical and/or patient‐centered outcomes	76 (79.2)
Assess the efficacy of deprescribing interventions to optimize prescribing outcomes (ie, reduce medication burden)	14 (14.6)
Generate evidence on medication harms	2 (2.1)
Generate evidence on medication efficacy	1 (1.0)
Other	3 (3.1)
Common barriers	
Time and effort required to conduct	53 (18.2)
Establishing and/or maintaining relationships with other health professionals involved in patient care	50 (17.1)
Incorporating patients’/carers’ opinions on the deprescribing process	37 (12.7)
Coordinating deprescribing process for the patient	37 (12.7)
Coordinating logistics of deprescribing in the setting	35 (12.0)
Harnessing practitioner's skills and knowledge into the deprescribing process	30 (10.3)
Obtaining adequate patient consent	23 (7.9)
Funding	16 (5.5)
Other	11 (3.8)
Common enablers	
Beliefs of other health professionals regarding benefits of deprescribing	57 (24.4)
Willingness of patients to participate	49 (20.9)
Researcher, health professional, and/or patient experience with deprescribing	46 (19.7)
Support from staff at recruitment sites	44 (18.6)
Previous experience of people involved with conducting deprescribing trials	25 (10.7)
Other	13 (5.6)

Note

Participants were able to select more than one option for barriers and enablers; N = 96.

### 4.2 Pretrial approval and participant recruitment barriers

In seeking ethical approval for deprescribing trials (Table 3), respondents indicated the primary barriers were “the recruitment of vulnerable participants” (18.9%) or those “unable to provide verbal or written consent” (18.5%). When attempting to seek national regulatory authority approval, a major barrier was “establishing and demonstrating safe implementation of… intervention” (41.5%). When recruiting participants into clinical deprescribing trials, respondents indicated the major barriers were “beliefs and opinions of health professionals… influencing the decision” (31.0%) and “recruitment of participants who are unable to consent” (27.7%).

**Table 3:** Responses to questions surveying pretrial and recruitment barriers in clinical deprescribing trials

Question theme	Number of responses (%)
Major barriers to ethical approval	
Recruitment of vulnerable participants	47 (18.9)
Recruitment of participants who are unable to provide verbal or written consent (ie, access to carer issues)	46 (18.5)
Seeking approval for ethics from multiple stakeholders, for example, nursing home, nursing staff, and patient	42 (16.9)
Ethics committee inexperience with reviewing protocols for deprescribing trials	41 (16.5)
Potential for adverse drug withdrawal events associated with deprescribing medications	35 (14.1)
Limited evidence of benefit of deprescribing medications	27 (10.8)
Other	11 (4.4)
Major barriers to national regulatory authority approval	
Establishing and demonstrating safe implementation of a multidisciplinary deprescribing intervention	54 (41.5)
Demonstrating good clinical practice according to national directives	26 (20.0)
Demonstrating quality assurance systems	21 (16.2)
Establishing and demonstrating safe manufacturing of placebo and study drug(s)	12 (9.2)
Do not know/did not understand question/not applicable	10 (7.7)
Passing audits and inspections	2 (1.5)
Other	5 (3.8)
Major recruitment barriers	
Beliefs and opinions of health professionals caring for their patients influencing the decision to deprescribe treatments	57 (31.0)
Recruitment of participants who are unable to consent during the screening process due to external factors—for example, carer not present, too ill	51 (27.7)
Patient and/or carer apprehension	37 (20.1)
Co‐ordination of study organization between researcher, recruiter and patient and/or carer, and their treating health professionals	30 (16.3)
Other	9 (4.9)

Note

Participants could select more than one option; N = 96.

### 4.3 Appropriateness of clinical trial methodologies and participant recruitment sites

**Figure 2:**
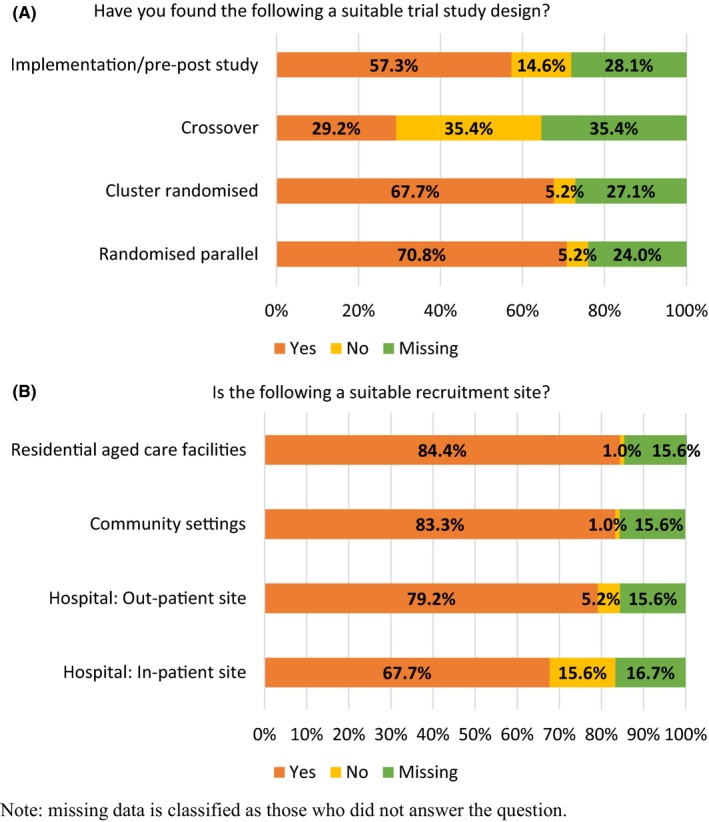
Responses to questions about the appropriateness of using different trial methodologies (2A) and settings (2B) to conduct clinical deprescribing trials

In determining appropriate study methodologies and sites (Figure 2) respondents indicated most classical trial designs were suitable (79.7%‐93.2%). Classical parallel RCTs were considered the most appropriate (93.2 ± 6.0%) vs “crossover” studies (45.2 ± 12.4%). Respondents indicated this was because it would not be safe to restart potentially harmful drugs. All options for study recruitment sites (hospital in and out‐patients, nursing homes, and community settings) were deemed appropriate (81.3%‐98.8%), although hospital in‐patient sites were considered the least appropriate (81.3 ± 9.8%) due to a heterogeneous patient group and time constraints.

### 4.4 Potential need for future framework and CONSORT list amendment

**Figure 3:**
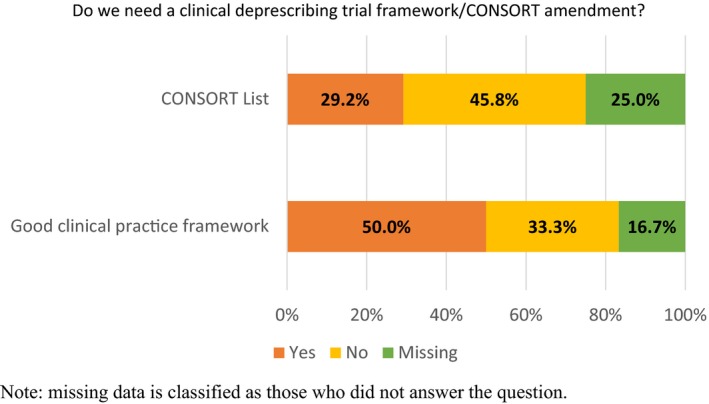
Responses to questions about future clinical deprescribing trial framework and CONSORT list amendment to include clinical deprescribing trials

Finally, respondents were asked if they believed a “legal, regulatory and good clinical practice framework” needed to be developed for clinical deprescribing trials, and whether the CONSORT list required amending to include deprescribing trials (Figure 3). Most respondents indicated that a good clinical practice framework did need to be developed (60.0 ± 11.0%), but that the current CONSORT list did not need amending (38.9% yes ± 11.6%). There was greater resistance to amending the CONSORT list if participants had previously conducted clinical deprescribing trials compared to those who had not been involved in clinical deprescribing trials (70.3% vs 51.4%, respectively), although this was not significant (Table 4). There was little difference in support levels for a clinical deprescribing framework based on previous clinical deprescribing trial experience (59.0% experienced vs 61.0% no experience) (Table 4).

**Table 4:** Responses to questions about a future clinical deprescribing trial framework and CONSORT list amendment to include clinical deprescribing trials, based on previous experience in clinical deprescribing trials

Question		Have you previously been involved in clinical deprescribing trials?	
		Yes (%)	No (%)
Do you think we need to develop a legal, regulatory, and good clinical practice framework for clinical deprescribing trials?	Yes	61.0	39.0
	No	59.0	41.0
Does the current CONSORT list need to be amended to include clinical deprescribing trials?	Yes	48.6	51.4
	No	29.7	70.3

Common themes emerging from respondents’ open‐ended answers revealed that a clinical deprescribing trial framework should ensure constant safety monitoring, and include robust and clinically significant outcome measures. However, those who did not believe a framework was required, nor that the CONSORT list needed amending, felt that there is already too much red tape in conducting clinical deprescribing trials, and that studies can be conducted under current frameworks (8.3% of respondents who gave comments to both of the final two questions). The full‐text comments to the last two questions are in the Supplementary Information.

## 5 DISCUSSION

To our knowledge, this is the first study that has systematically investigated the perspectives, attitudes, interests, barriers, and enablers in relation to conducting clinical deprescribing trials among health professionals and researchers internationally.

Respondents cited the main rationale for conducting deprescribing studies is to optimize patient‐centered outcomes, and indicated that the positive beliefs of participants and their treating clinicians regarding deprescribing were the greatest enablers. The most common barriers were the time and effort required for a clinical deprescribing trial, and the apprehension toward deprescribing of a health professional involved in the study, especially if they are involved in participant recruitment. Finally, respondents specified that classical RCTs are the most appropriate trial methodology to employ and should form the backbone of any good clinical practice framework. The attitudes, interests, barriers, and enablers of conducting clinical deprescribing trials did not alter whether respondents had previously conducted studies or not.

The primary concern reported by health professionals regarding deprescribing is safety.^5^, ^12^, ^18^, ^20^, ^21^, ^22^, ^25^ Ensuring patient safety should be the primary factor in influencing any decision made by a treating clinician—but often there is uncertainty on what deprescribing involves and its potential benefits; especially in vulnerable patient groups.^12^ These vulnerable patient groups, including those “where consent must be acquired through a proxy”, and those who “transition through various healthcare settings”, were recognized as the most difficult patients to enroll and retain in clinical deprescribing trials (39.5% and 44.2%, respectively). This challenge extends to when institutional ethical approval is sought, with the most commonly identified barrier being the recruitment of “vulnerable participants”, and those “unable to provide… consent” (18.9% and 18.5%, respectively).

Previous studies, however, have demonstrated that patients, while having strong preexisting attitudes toward drug use, are willing to cease treatments if there may be a positive health benefit, they can reduce their drug burden, and it is deemed appropriate by their doctor.^19^, ^23^, ^26^ This is reflected with the strong response on the “willingness of patients to participate” (20.9%) as an enabler in conducting clinical deprescribing trials. Indeed, a recent study concluded that older adults generally fall into three categories regarding attitudes toward drugs and overall opinions on deprescribing: type 1 who are positive toward drugs and leave decisions to their doctors; type 2 who are more proactive and open to deprescribing; and type 3 who are generally frail and defer decisions to their doctor or caregiver.^26^ To utilize these beliefs, and ensure adequate participant recruitment, patients, and care givers, must be presented with evidence on the potential benefits of deprescribing, and how patient safety will be ensured.

To overcome prescribers’ and participants’ fears, a rigorous trial methodology must be practiced and respondents strongly indicated that parallel RCTs (93.2%) and cluster RCTs (92.9%) were the most appropriate trial methods for clinical deprescribing trials. Implementation/pre‐post studies were also deemed by most respondents as appropriate (79.7%), although crossover trials were deemed as not appropriate (54.8%). These attitudes toward crossover trials are reflected in current literature; with a recent narrative review finding that of 33 randomized controlled trials deprescribing single drugs, 32 employed a parallel design.^30^ Also, this review supported the assertions of this survey's respondents, in deeming that community and residential aged care settings (98.8% each) are the most suitable recruitment sites, with only four studies recruiting from other sites.

Despite the popularity of using RCTs in deprescribing research, some studies have incorporated a crossover methodology for deprescribing, with a systematic review identifying six such trials.^31^ These studies generated “strong patient‐specific evidence” on the effectiveness of treatments while an RCT may only have captured the cohort benefit‐to‐harm response. However, while RCTs, and N‐of‐1 trials, have been utilized in small deprescribing trials, on larger scales they can often be expensive (another identified barrier [5.5%]). Other researchers have performed retrospective time series analysis in real‐world populations following the implementation of a deprescribing intervention. One study improved the use of proton‐pump inhibitors (PPIs) in Australian veterans by distributing various educational materials to patients and prescribers.^32^ Overall effect was a 20.9% decrease in PPI use and 42.2% increase in lower‐dose PPI use 12 months after the final intervention. This study indicates that a variety of methods can be used in deprescribing trials to progress and translate research from the clinic to populations. Any strategies that are employed, however, need to cover the four primary safety concerns of deprescribing: adverse drug withdrawal events; return of medical condition(s); reversal of drug‐drug interactions; and, damage to the doctor‐patient relationship.^12^

Considering the safety concerns of health professionals treating patients, the willingness of patients to participate, and the reported lack of strong evidence, it is surprising that 40% of respondents indicated that a “good clinical practice framework for clinical deprescribing trials” was not required. Also, 61.1% of respondents indicated that the current CONSORT list did not need to be amended. Respondents thought that any framework or CONSORT list modification would increase the amount of red tape that already surrounds deprescribing trials. Also, respondents felt that there are already sufficient good clinical practice frameworks and that deprescribing trials can be conducted under current CONSORT extensions. However, of those who did indicate the need for a “good clinical practice framework”, the most common suggestion was to ensure constant and rigorous safety monitoring for ADEs, adverse drug withdrawal events, and potential rebound of disease. Any framework should consider that deprescribing trials can be conducted within a wide range routine clinical care with patient‐specific outcomes related to individual or collective health goals.

Our findings indicate there is already a strong interest in deprescribing trials, with the developing body of research allowing parties to support their interests and conduct their own studies. As the evidence base grows of the benefits and harms of drugs in different populations, studies can be conducted where there remains genuine uncertainty about the role of deprescribing. Furthermore, our study findings are consistent with many of the ideas and beliefs brought forward in the World Café discussion were reflected in the survey results.^12^, ^24^ Primarily, the need for more clinical deprescribing trials that focus on and utilize patient‐centered clinical interventions and outcomes, an area that was considered of utmost importance. Also, the researchers recognized the requirement of engaging other health professionals involved in the deprescribing process to improve and tailor interventions and outcomes, as well as the need to perform clinical deprescribing trials in “high‐risk” patient groups.

### 5.1 Strengths and limitations

The main strength was that this survey was informed by international and national experts outside the research team and was distributed to an international audience encompassing a variety of occupations and experience levels in deprescribing trials. The high participation rate suggests that the data are representative of those to whom it was administered, namely researchers and health professionals interested and involved in clinical deprescribing trials. Finally, the survey was completely anonymous, minimizing bias when interpreting results.

The survey did have some limitations. Some respondents indicated that some questions were not applicable to them, especially regarding national regulatory authorities that may not be present in their jurisdiction (10.4%). Also, the survey was targeted at people already interested in deprescribing, limiting the generalizability of the findings to a wider audience, although not all respondents had experience in conducting deprescribing studies. Future researchers could use the findings from this survey and readminister it to other health professionals to discover if the attitudes are reflective of a greater community. Additionally, the impact of response shift was not captured in this survey, where beliefs and opinions toward different methods and factors of conducting clinical deprescribing trials may alter based on the lived experiences of respondents. However, given there were no differences in responses whether respondents had previously conducted clinical deprescribing trials or not, response shift may not be a large factor in participants’ responses. Finally, it was not possible to determine the exact response rate from the due to not knowing how many potential participants received the survey within each organization mailing list.

In conclusion, researchers and health professionals had a variety of opinions on conducting clinical deprescribing trials with some key themes emerging. Our findings suggest that deprescribing trials should be conducted to optimize patient‐centered outcomes with health professional engagement of paramount importance to ensure the conduct of a clinical deprescribing trial and enable evidence synthesis across trials. Future studies should ideally establish a clinical deprescribing trial framework with RCTs as a model, which emphasizes monitoring safe clinical practice at all stages and targets patient‐centered outcomes. The findings and recommendations of this study could also be presented to other health care professionals not engaged in deprescribing, to gather their opinions on conducting clinical deprescribing trials and inform future researchers.

## ETHICS APPROVAL AND CONSENT TO PARTICIPATE

Ethics approval was granted by the University of Sydney's Human Research Ethics Committee, project number [2017/295]. The first page of the survey was the Participant Information Statement, indicating that submission of the completed survey was an indication of consent to participate in this study.

## DISCLOSURE

There are no competing interests to declare.

## AUTHORS’ CONTRIBUTIONS

AJC designed, revised, and distributed the survey, monitored data for the whole trial, wrote the statistical analysis plan, collected, cleaned and analyzed the data, and drafted and revised the paper. He is the guarantor. DG designed, revised and distributed the survey, monitored data collection, analyzed the data, and revised the paper. SNH revised the survey, analyzed the data, and revised the paper. LK revised the survey, analyzed the data, revised the statistical analysis plan, and revised the paper. SLN revised the paper.

## References

[1] Page A , Clifford R , Potter K , Etherton‐Beer C . A concept analysis of deprescribing medications in older people. J Pharm Pract Res. 2018;48(2):132‐148.

[2] Beer C , Loh PK , Peng YG , Potter K , Millar A . A pilot randomized controlled trial of deprescribing. Ther Adv Drug Saf. 2011;2(2):37‐43. 25083200 10.1177/2042098611400332 PMC4110808

[3] Gnjidic D , Le Couteur DG , Kouladjian L , Hilmer SN . Deprescribing trials: methods to reduce polypharmacy and the impact on prescribing and clinical outcomes. Clin Geriatr Med. 2012;28(2):237‐253. 22500541 10.1016/j.cger.2012.01.006

[4] Naunton M , Peterson GM , Deeks LS , Young H , Kosari S . We have had a gutful: the need for deprescribing proton pump inhibitors. J Clin Pharm Ther. 2018;43(1):65‐72. 28895169 10.1111/jcpt.12613

[5] Ailabouni NJ , Nishtala PS , Mangin D , Tordoff JM . Challenges and enablers of deprescribing: a general practitioner perspective. PLoS ONE. 2016;11(4):e0151066. 27093289 10.1371/journal.pone.0151066 PMC4836702

[6] Boghossian TA , Rashid FJ , Thompson W , et al. Deprescribing versus continuation of chronic proton pump inhibitor use in adults. Cochrane Database Syst Rev. 2017;3:Cd011969. 28301676 10.1002/14651858.CD011969.pub2 PMC6464703

[7] Hilmer SN , Gnjidic D , Le Couteur DG . Thinking through the medication list ‐ appropriate prescribing and deprescribing in robust and frail older patients. Aust Fam Physician. 2012;41(12):924‐928. 23210113

[8] Pollmann AS , Murphy AL , Bergman JC , Gardner DM . Deprescribing benzodiazepines and Z‐drugs in community‐dwelling adults: a scoping review. BMC Pharmacol Toxicol. 2015;16:19. 26141716 10.1186/s40360-015-0019-8 PMC4491204

[9] Reeve E , Denig P , Hilmer SN , Ter Meulen R . The ethics of deprescribing in older adults. J Bioeth Inq. 2016;13(4):581‐590. 27416980 10.1007/s11673-016-9736-y

[10] Donaldson LJ , Kelley ET , Dhingra‐Kumar N , Kieny MP , Sheikh A . Medication without harm: WHO's third global patient safety challenge. Lancet. 2017;389(10080):1680‐1681. 28463129 10.1016/S0140-6736(17)31047-4

[11] Farrell B , Tsang C , Raman‐Wilms L , Irving H , Conklin J , Pottie K . What are priorities for deprescribing for elderly patients? Capturing the voice of practitioners: a modified delphi process. PLoS ONE. 2015;10(4):e0122246. 25849568 10.1371/journal.pone.0122246 PMC4388504

[12] Reeve E , Moriarty F , Nahas R , Turner JP , Kouladjian O'Donnell L , Hilmer SN . A narrative review of the safety concerns of deprescribing in older adults and strategies to mitigate potential harms. Expert Opin Drug Saf. 2018;17(1):39‐49. 29072544 10.1080/14740338.2018.1397625

[13] Renn BN , Asghar‐Ali AA , Thielke S , et al. A systematic review of practice guidelines and recommendations for discontinuation of cholinesterase inhibitors in dementia. Am J Geriatr Psychiatry. 2017;26(2):134‐147. 29167065 10.1016/j.jagp.2017.09.027 PMC5817050

[14] CaDeN . Deprescribing Algorithms; 2017 Retrieved 20 September, 2017, from https://www.deprescribingnetwork.ca/algorithms.

[15] Farrell B , Pottie K , Rojas‐Fernandez CH , Bjerre LM , Thompson W , Welch V . Methodology for developing deprescribing guidelines: using evidence and GRADE to guide recommendations for deprescribing. PLoS ONE. 2016;11(8):e0161248. 27517450 10.1371/journal.pone.0161248 PMC4982638

[16] Reeve E , Thompson W , Farrell B . Deprescribing: a narrative review of the evidence and practical recommendations for recognizing opportunities and taking action. Eur J Intern Med. 2017;38:3‐11. 28063660 10.1016/j.ejim.2016.12.021

[17] Tasmania PH . Deprescribing. June 7, 2016 Retrieved 20 September, 2017, from https://www.primaryhealthtas.com.au/resources/deprescribing.

[18] Anderson K , Foster M , Freeman C , Luetsch K , Scott I . Negotiating “Unmeasurable Harm and Benefit”: perspectives of general practitioners and consultant pharmacists on deprescribing in the primary care setting. Qual Health Res. 2017;27(13):1936‐1947. 29088989 10.1177/1049732316687732

[19] Clyne B , Cooper JA , Boland F , Hughes CM , Fahey T , Smith SM . Beliefs about prescribed medication among older patients with polypharmacy: a mixed methods study in primary care. Br J Gen Pract. 2017;67(660):e507‐e518. 28533200 10.3399/bjgp17X691073 PMC5540192

[20] Kouladjian L , Gnjidic D , Reeve E , Chen TF , Hilmer SN . Health care practitioners’ perspectives on deprescribing anticholinergic and sedative medications in older adults. Ann Pharmacother. 2016;50(8):625‐636. 27257284 10.1177/1060028016652997

[21] Linsky A , Meterko M , Stolzmann K , Simon SR . Supporting medication discontinuation: provider preferences for interventions to facilitate deprescribing. BMC Health Serv Res. 2017;17(1):447. 28659157 10.1186/s12913-017-2391-0 PMC5490086

[22] Ni Chroinin D , Ni Chroinin C , Beveridge A . Factors influencing deprescribing habits among geriatricians. Age Ageing. 2015;44(4):704‐708. 25758409 10.1093/ageing/afv028

[23] Qi K , Reeve E , Hilmer SN , Pearson SA , Matthews S , Gnjidic D . Older peoples’ attitudes regarding polypharmacy, statin use and willingness to have statins deprescribed in Australia. Int J Clin Pharm. 2015;37(5):949‐957. 26047944 10.1007/s11096-015-0147-7

[24] Thompson W , Reeve E , Moriarty F , et al. Deprescribing: future directions for research. Res Social Adm Pharm. 2018 10.1016/j.sapharm.2018.08.013 PMC6422759 30241876

[25] Wallis KA , Andrews A , Henderson M . Swimming against the tide: primary care physicians’ views on deprescribing in everyday practice. Ann Fam Med. 2017;15(4):341‐346. 28694270 10.1370/afm.2094 PMC5505453

[26] Weir K , Nickel B , Naganathan V , et al. Decision‐making preferences and deprescribing: perspectives of older adults and companions about their medicines. J Gerontol B Psychol Sci Soc Sci. 2018;73:e98‐e107. 29190369 10.1093/geronb/gbx138

[27] Gnjidic D , Le Couteur DG , Hilmer SN . Discontinuing drug treatments. BMJ. 2014;349:g7013. 25416561 10.1136/bmj.g7013

[28] Eysenbach G . Improving the quality of Web surveys: the Checklist for Reporting Results of Internet E‐Surveys (CHERRIES). J Med Internet Res. 2004;6(3):e34. 15471760 10.2196/jmir.6.3.e34 PMC1550605

[29] Hsieh HF , Shannon SE . Three approaches to qualitative content analysis. Qual Health Res. 2005;15(9):1277‐1288. 16204405 10.1177/1049732305276687

[30] Page AT , Potter K , Clifford R , Etherton‐Beer C . Deprescribing in older people. Maturitas. 2016;91:115‐134. 27451330 10.1016/j.maturitas.2016.06.006

[31] Clough AJ , Hilmer SN , Naismith SL , Kardell LD , Gnjidic D . N‐of‐1 trials for assessing the effects of deprescribing medications on short‐term clinical outcomes in older adults: a systematic review. J Clin Epidemiol. 2017;93:112‐119. 28951110 10.1016/j.jclinepi.2017.09.015

[32] Pratt NL , Kalisch Ellett LM , Sluggett JK , et al. Use of proton pump inhibitors among older Australians: national quality improvement programmes have led to sustained practice change. Int J Qual Health Care. 2017;29(1):75‐82. 27920248 10.1093/intqhc/mzw138

