# Supplementary material for: Health professionals’ and researchers’ opinions on conducting clinical deprescribing trials
Source: Pharmacol Res Perspect. 2019 Apr 25;7(3):e00476. doi: 10.1002/prp2.476 (PMC6482940; doi:10.1002/prp2.476)
Supplement: Supplementary file 1 [file PRP2-7-e00476-s001.docx]

**SUPPLEMENTARY INFORMATION**

**Methods: Emailed Letters of Invitation**

**Letter of Invitation – Individual Researcher**

Dear __________________ (individual researcher)

You have been invited to participate in a survey which aims to capture researchers’ and health professionals’ perspectives and attitudes in relation to conducting clinical deprescribing trials

You have been invited as you have conducted clinical deprescribing trials and we believe your experience and opinions on deprescribing research and trials would give us a valuable insight into this field and potential future directions.

The survey will take approximately 10-15 minutes to complete. Your individual response will be confidential. Your identity as a participant will remain anonymous.

If you have any concerns or complaints about the ethical conduct of the research project, you may contact the Ethics Administration Manager, University of Sydney +61 2 8627 8176 [human.ethics@sydney.edu.au](mailto:human.ethics@sydney.edu.au) (Study number XXXX-XXX)

If you would like to continue to participate in the study, please use the survey link below to enter and complete the survey online. Please note that completion and submission of this survey means that you consent to participate in this study.

Please complete the survey only once.

On behalf of the study team, we thank you for your time in considering this study.

Yours Sincerely,

Mr Alexander Clough, Research Student

Dr Danijela Gnjidic, Research Fellow and Lecturer

Faculty of Pharmacy, University of Sydney

**Letter of Invitation – Organisation Member**

Dear (organisation) member

You are invited to participate in a survey which aims to capture researchers’ and health professionals’ perspectives and attitudes in relation to conducting clinical deprescribing trials

You have been invited as you are a member of ___________ (organisation) and we believe your experience and opinions on deprescribing research and trials would give us a valuable insight into this field and potential future directions.

The survey will take approximately 10-15 minutes to complete. Your individual response will be confidential. Your identity as a participant will remain anonymous.

If you have any concerns or complaints about the ethical conduct of the research project, you may contact the Ethics Administration Manager, University of Sydney +61 2 8627 8176 [human.ethics@sydney.edu.au](mailto:human.ethics@sydney.edu.au) (Study number XXXX-XXX)

If you would like to continue to participate in the study, please use the survey link below to enter and complete the survey online. Please note that completion and submission of this survey means that you consent to participate in this study.

Please complete the survey only once.

On behalf of the study team, we thank you for your time in considering this study.

Yours Sincerely,

Mr Alexander Clough, Research Student

Dr Danijela Gnjidic, Research Fellow and Lecturer

Faculty of Pharmacy, University of Sydney

**Methods: Survey Questions**

**Information:**

1. What is your age? _______________________________________________
2. What is your gender?
   1. Male
   2. Female
3. What country are you based in? ____________________________________
4. What is your current occupation?
   1. Academic
   2. Physician
   3. Health Professional (please define) ____________________________
   4. Student Researcher
   5. Other (please specify) ______________________________________
5. Have you previously been involved in deprescribing trials?
   1. Yes
   2. No
6. What is your primary research area? ____________________________________________________________________________________________________________________________

**The following 3 questions are about the role of deprescribing trials in public health, and the major barriers and enablers of conducting these trials. All responses are completely anonymous.**

TRIAL CONCEPT

1. In your opinion, what is the main rationale for conducting deprescribing clinical trials? Is it to:
   1. Generate evidence on medication efficacy
   2. Generate evidence on medication harms
   3. Assess the efficacy of deprescribing interventions to optimise prescribing outcomes (i.e. reduce medication burden)
   4. Assess the efficacy of deprescribing interventions to optimise clinical and/or patient centred outcomes
   5. Other (please specify) ______________________________________

BARRIERS AND ENABLERS

1. In your opinion, what are the common barriers when conducting clinical deprescribing trials? Select more than one if necessary.
   1. Obtaining adequate patient consent
   2. Time and effort required to conduct deprescribing trials
   3. Co-ordinating the deprescribing process for the patient (e.g. dates of assessments, adhering to protocol)
   4. Co-ordinating logistics of deprescribing in the setting
   5. Establishing and/or maintaining relationship with other health professionals involved in patient care
   6. Incorporating patients/carers opinions on the deprescribing process
   7. Harnessing the practitioner’s skills and knowledge into the deprescribing process
   8. Other (please specify – e.g. work setting barriers, funding) ________________________________________________________
2. In your opinion, what are the common enablers when conducting clinical deprescribing trials? Select more than one if necessary.
   1. Willingness of patients to participate
   2. Beliefs of health professionals regarding benefits of deprescribing
   3. Support from staff at recruitment sites
   4. Researcher, health professional and/or patient experience with deprescribing
   5. Previous experience of people involved with conducting deprescribing studies
   6. Other (please specify – e.g. work setting enablers, willingness of patients to participate) ________________________________________________________

**The next 3 questions are about ethical issues of conducting and the design of clinical deprescribing trials**

ETHICS ISSUES

1. In your opinion, what are the major barriers to obtaining ethics approval for deprescribing trials?
   1. Recruitment of vulnerable participants (e.g. older adults, presence of co-morbidities)
   2. Recruitment of participants who are unable to provide verbal or written consent (i.e. access to carer issues)
   3. Ethics committee inexperience with reviewing protocols for deprescribing trials
   4. Limited evidence of benefit of deprescribing medications
   5. Potential for adverse drug withdrawal events associated with deprescribing medications
   6. Seeking approval for ethics from multiple stakeholders e.g. nursing home, nursing staff, patient
   7. Other (please specify – e.g. time constraints) ________________________________________________________
2. In your opinion, what are the main challenges when gaining approval from the national regulatory authority do conduct deprescribing trials?
   1. Demonstrating good clinical practice according to national directives
   2. Demonstrating quality assurance systems
   3. Passing audits and inspections
   4. Establishing and demonstrating safe manufacturing of placebo and study drug(s)
   5. Establishing and demonstrating safe implementation of a multidisciplinary deprescribing intervention
   6. Other (please specify – e.g. hidden/unknown red tape) ________________________________________________________

CLINICAL TRIAL DESIGN

1. In your opinion, which trial study design have you found to be appropriate when conducting deprescribing trials?

| Clinical Trial Design | Appropriate? | | If not appropriate, why? |
| --- | --- | --- | --- |
|  | Yes | No |  |
| Randomised parallel – discontinue or continue |  |  |  |
| Cluster randomised |  |  |  |
| Crossover |  |  |  |
| Implementation/pre-post study |  |  |  |

**The next 4 questions are about the recruitment stage and organisation of clinical deprescribing trials**

PARTICIPANT RECRUITMENT

1. In your opinion, which patients are the most difficult to enrol and retain in deprescribing trials?
   1. People aged 50 and above
   2. People with chronic conditions
   3. People with specific conditions such as mental health issues where consent must be acquired from surrogate/proxy decision makers
   4. “Frequent flyer” patients who transition through various healthcare settings
   5. Other (please specify – e.g. children, people exposed to polypharmacy) ________________________________________________________
2. In your opinion, what are the major barriers in the process of recruiting participants into deprescribing trials?
   1. Recruitment of participants who are unable to consent during the screening process due to external factors – e.g. carer not present, too ill
   2. Beliefs and opinions of health professionals caring for their patients influencing the decision to deprescribe treatments
   3. Patient and/or carer apprehension
   4. Co-ordination of study organisation between researcher, recruiter and patient and/or carer, and their treating health professionals
   5. Other (please specify – e.g. time constraints) ________________________________________________________
3. In your opinion, which of the following suitable recruitment sites for deprescribing trials?

| Recruitment Site | Suitable | | If not suitable, why? |
| --- | --- | --- | --- |
|  | Yes | No |  |
| Hospital: In-patient site |  |  |  |
| Hospital: Out-patient clinics |  |  |  |
| Community settings (e.g. GP clinics) |  |  |  |
| Residential aged care facilities |  |  |  |

STUDY ORGANISATION

1. In your opinion, what are the major factors in facilitating the effective completion of clinical deprescribing trials in general?
   1. Effective patient screening process
   2. Effective communication between health professionals caring for patients, patients, and researchers
   3. Managing prescribers’ fears about deprescribing medications and devolving responsibility for any adverse events
   4. Ensuring intervention type is appropriate for the study and outcomes being investigated – e.g. pharmacological vs non-pharmacological interventions, or single vs multiple drug cessation trials
   5. Other (please specify) ______________________________________

**The final 2 questions will explore the potential for developing a clinical deprescribing trial framework**

1. Do you think we need to develop a legal, regulatory, and good clinical practice framework for clinical deprescribing trials?
2. Yes, and the 3 key components should be:
   1. ____________________________________________________________________________________________________________
   2. ____________________________________________________________________________________________________________
   3. ____________________________________________________________________________________________________________
3. No, because:
   1. **____________________________________________________________________________________________________________**
   2. **____________________________________________________________________________________________________________**
   3. **____________________________________________________________________________________________________________**
4. Does the current CONSORT list [[http://www.consort-statement.org/consort-statement/checklist](http://www.consort-statement.org/consort-statement/checklist%20)] need to be amended to include deprescribing trials?
   1. Yes, and the 3 key inclusions to differentiate clinical deprescribing trials from other trial checklists should be:
      1. **__________________________________________________________________________________________________________**
      2. **__________________________________________________________________________________________________________**
      3. **__________________________________________________________________________________________________________**
   2. No, because:
      1. __________________________________________________________________________________________________________
      2. __________________________________________________________________________________________________________
      3. __________________________________________________________________________________________________________

**This is the end of the survey, thank you very much for your responses.**

**Would you like to receive feedback about the overall results of this study?**

**YES** 🞏 **NO** 🞏

If you answered **YES**, please nominate your preferred email address.

🞏 Email: __________________________________________________

**If you have any questions or queries, feel free to contact myself, Mr Alexander Clough at** [**aclo4212@uni.sydney.edu.au**](mailto:aclo4212@uni.sydney.edu.au)**, or Dr Danijela Gnjidic at** [**danijela.gnjidic@sydney.edu.au**](mailto:danijela.gnjidic@sydney.edu.au)**.**
