# Supplementary material for: Health professionals’ and researchers’ opinions on conducting clinical deprescribing trials
Source: Pharmacol Res Perspect. 2019 Apr 25;7(3):e00476. doi: 10.1002/prp2.476 (PMC6482940; doi:10.1002/prp2.476)
Supplement: Supplementary file 2 [file PRP2-7-e00476-s002.docx]

**Supplementary Information:**

**Results: Full survey comments on the requirement for the development of a good clinical practice framework and CONSORT list amendment, as perceived by health professionals and researchers**

Question 11: Full-text responses to respondents answering yes

| Response (1=Yes, 2=No) | Comments | | | |
| --- | --- | --- | --- | --- |
| 1 | Provision of a well-detailed protocol (explaining for example how each step will be performed, what will be done specifically in case a deprescribing step for such and such medication fails, etc) | | Responsibilities of the researchers (qualifications required, data management, legal and ethical issues, knowledge transfer) | |
| 1 | Applicable in daily practice | Responding to the needs and preferences of involved patients and their their carer(s) and family. | | Corresponding with patient-related outcomes |
| 1 | Rigorous safety monitoring | Appropriate systems and processes in place to manage ADE/ADWE | | Ensuring appropriate (representative) populations are involved including older people, dementia etc |
| 1 | Patient involvement (shared-decision making) | |  | |
| 1 | Figuring out how studies can be done pragmatically and in routine care (not highly selected typical efficacy trial) | what outcomes to measure | | ethical issues |
| 1 | status of placebo encapsulated tablets | expert panel to review | | consent issues in people without capacity |
| 1 | Evidence based guidelines for deprescribing where there is trial evidence | Guidance for deprescribing where there is little or no evidence | | a governance framework to work to when deprescribing to assure staff of the safety and appropriateness of deprescribing and provide assurance to patients of the benefits of appropriate deprescribing |
| 1 | If a medication is stopped and the patient does not get any benefit (e.g. does not reduce hospital readmissions, does not reduce number of falls etc), however there is no harm, then this should be perceived as a benefit in itself | | All medications with a high potential to cause rebound symptoms should be weaned- even if the doctor might want to stop it right away, by weaning it'll reduce risk of adverse drug withdrawal events. if it is stopped abruptly and the patient experiences rebound symptoms, then this will discourage them from reviewing the medication in the future | |
| 1 | Establish proper methodology (ie RCT, blinding) | Suggesting processes for appropriate deprescription and assessment of potential side effects, specially for multiple drugs | | Suggesting comparable otucomes that are relevant for participants |
| 1 | Guidance regarding study design | Templates for regulatory approval | | Guidelines on ethical considerations |
| 1 | I'm not totally sure how to respond | |  | |
| 1 | safety | efficacy | | cost-savings |
| 1 | adequate follow up to better enroll 'on the fence' patients | |  | |
| 1 | Ensure patient safety | Engage all health professionals | | Improve delivery of deprescribing trials |
| 1 | Trial registration, Full disclosure of trial results to all participants within 3 months of close of trial, required reporting within 1 year. | | | |
| 1 | Safe: Patient safety is the number one priority. Systems must be in place to protect and monitor the patient | Robust: Well-designed so that the evidence is useful and usable by end users | | Patient centered: Should consider and respect the values and preferences and context of the patient. May require de-prescribing to more be more pragmatic and less explanatory in nature |
| 1 | I have insufficient knowledge to answer the question | I have insufficient knowledge to answer the question | | I have insufficient knowledge to answer the question |
| 1 | A broad statement defining eligibility for de-prescribing that can assist clinicians and researchers to identify potential candidates. | | Protections against risks of de-prescribing. | |
| 1 | Yes because without rules, there will be no change and the professionnals work with the good clinical practice | yes to force the health professionals to change their practice. | | To supervise and facilitate their work |
| 1 | specific indicators | matched group | | timing consistent |
| 1 | Health variables to be monitored after deprescribing must be clearly defined in the protocol of the study | Follow up of the patients must be ensured in medical visits scheduled. | | Interventions made at the end of the life of a patient must be in agreement with his/her vital wills |
| 1 | Definitions (what is meant by deprescribing) | The best practices for deprescribing and how to follow those in a trial | | Supports for the staff involved (e.g. liability, responsibilities) |
| 1 | Definition of accepted and relevant health outcomes | Minimal requirements for patient selection and patient safety | | Minimal requirements for duration of follow-up and other methodological standards (accepted study type, statistical analyses and power etc) |
| 1 | safety | evidence | | collaborations |
| 1 | Patient safety | Consent | | Best practice |
| 1 |  |  | | a priori definitions should be created so approaches can be consistent (which also allows them to be more easier compared using meta-analysis techniques) |
| 1 | general rules | safety | | exclusion criteria to some pathology for wich treatment is indicated (example: bipolar disorders) |
| 1 | Liability risk | methods and strategies for communicating with patients and caregivers | | More Deprescribing protocols with supporting information |
| 1 | Legal | Regulatory | | Clinics |
| 1 | DEPRESCRIBING SHOULD BE PRIORITIZED WHEREVER A GOOD EVIDENCE FOR PRESCRIBING IS LACKING. ('DO NO HARM') | | MEANINGFUL OUTCOME LARGE-SCALE RANDOMIZED CONTROL TRIALS OF UNSOUND MEDICAL SYSTEM PRACTICES - SHOULD BE ENABLED BY A WAIVER OF PATIENT INFORMED CONSENT. | |
| 1 | A recommendation for the collection of clinically important outcome measures (e.g. falls resulting in fractures, hospitalisations, QOL) that will allow meta analysis of smaller trials. | | Guidelines from professional bodies to confirm deprescribing is part of 'good clinical practice,' minimising prescriber liability for potential ADWE's that may occur. | |
| 1 | EDUCATION | TIME | | EVIDENCE FOR HELAH & ECONOMIC BENEFITS FOLLOWING DEPRESCRIBING |
| 1 | collaboration across sites | communication | | interdisciplinary input |

| 2 | *there already is an EC per hospital/region/university. *we are in urgent need of data *Helsinki declaration is sufficient |
| --- | --- |
| 2 | Deprescribing as a potentially effective intervention is no different from other clinical interventions in terms of ethical requirements |
| 2 | Regulatory framework similar to prescribing sudies. |
| 2 | can be undertaken with current trial frameworks |
| 2 | Ceasing medicines is undertaken every day in clinical practice - however it is not systematic like screening. Eg Gps will regularly screen patients for high blood pressure but will not regularly screen them for deprescribing. A good clinical practice framework is required but I do not see that legal or regulatory action is required |
| 2 | first need a critical mass of studies |
| 2 | more red tape. Difficulty foreseeing all the future trial designs - so framework might limit flexibility. Already legal, regulatory, ethics approval processes in place. |
| 2 | This will generate yet another list / protocol. I think it would be more important to improve shared decision-making with patients. |
| 2 | Too early to say- not sure that this is really a regulatory issue; GCP might be helpful but the overall issue relates to informed consent and shared decision making |
| 2 | I think the international landscape varies so much in terms of prescribing practices that this is not feasible |
| 2 | no need, we have to follow the existent guidelines |
| 2 | I am concerned any legal obligations and extra regulation would act as a disincentive to researchers and funders. It could also stifle innovation in trial design and de-prescribing practices. I would happily support a best practice guideline or framework. This should ideally include an obligation by healthcare providers to respect the wishes and decisions of informed volunteers who opt to withdraw medications, appropriate outcome measures , no expectation of patient blinding, avoiding designs that introduce systemic biases (e.g. before and after). |
| 2 | Deprescribing trials would become very difficult to recruit patients if subjected to an overly restrictive framework. |
| 2 | There are already rules and procedures for clinical trials |
| 2 | Deprescribing trials should follow the same framework as any other clinical trial. Consider previous and quite successful work in this area such as publication by Garfinkel et al which did not require its own legal or regulatory framework. |
| 2 | We do not have a consensus on these components, they need to remain somewhat flexible for us to identify what these might be |
| 2 | no, I believe what we have is sufficient. |
| 2 | legal, regulatory and clinical practice framework already exists. |
| 2 | Likely not going to articulate the needs of all various sites, stakeholders so will have limited use/value. |
| 2 | different approaches will work for different groups. |
| 2 | will restrict views and narrow assumptions about what a good deprescribing trial is. Should provide guidance but nothing legal or regulatory, and even a best practice framework should be flexible. |
| 2 | fgjg |
| 2 | the fundamental issues are the same - balance of benefits and risks |
| 2 | no different to any other trial |
| 2 | We already have GCP for clinical trials. We do need other standardised procedures similar to other clinical trial areas e.g. MACE for cardiovascular or major bleeding definition for anticoagulant trials |
| 2 | Too early at this stage |
| 2 | these studies do not need new rules. Deprescribing in an intervention that is to be investigated just as any other health intervention. |

Question 12: Full-text responses to respondents answering yes

| Response (1=Yes, 2=No) | Comments | | |
| --- | --- | --- | --- |
| 1 | documentation withdrawal reactions | documentation of numbers of medications before and after intervention | protocol for deprescribing |
| 1 | fascinating. I'm not sure but I agree it should be amended. | | |
| 1 | Most of the CONSORT statement is fine, but participant blinding should not be expected for deprescribing trials. The principle that people should know what medications they are taking and why trumps any theoretical academic gain in scientific rigour from blinding. It may still be possible to blind outcome adjudicators from the trial team but this aspect of design is grossly over-rated, often with a detrimental effect on trial feasibility, cost and volunteer experience. | | |
| 1 | again, I will let others respond to this | | |
| 1 | No time to familiarize myself with Consort list tonight so have no comment. | | |
| 1 | age | co-morbidities | evidence |
| 1 | Trial design: not all deprescribing trials can be randomized | There may be a need for specific interventions accompanying the deprescribing process. These should be defined. | It will be important to predefine outcomes which are relevant as well as feasible to achieve and measure. |
| 1 | not sure | not sure | not sure |
| 1 | exclusion criteria | safety rules | general rules in relation with scientific evidence in deprescribing |
| 1 | dfgjghj | dfgjgfhj | dfjggfhj |
| 1 | PATIENT CENTERED MEDICINE | ETHICS | PROMUM NON NOCERAE - FIRST DO NOT HARM |

| 2 | The checklist seems adequate for conducting deprescribing trials. |
| --- | --- |
| 2 | The list seems complete and comprehensive enough |
| 2 | I believe we need to see more RCTs. And for me, a RCT is a RCT, whether it concerns deprescribing practices or not. |
| 2 | As above for Q11 (no different from other clinical interventions) |
| 2 | Seems premature at this point |
| 2 | already easy enough to interpret deprescribing trials within existing CONSORT list. |
| 2 | The CONSORT criteria seem to suffice although monitoring clinicians' adherence to the intervention seems to lack. Global monitoring of adherence could be useful (a very detailed monitoring may frustrate the medication review process). I also tend to favor not to replace the expertise of elderly care physicians / geriatricians and/or their professional guidelines and standards by CONSORT criteria because you do not want to have drugs deprescribed by medical psychologists / monkeys |
| 2 | I don't think the area requires a specific extension - so long as the interventions for each group are described with sufficient details to allow replication, including how and when they were actually administered, as per the current CONSORT statement |
| 2 | meets requirements |
| 2 | the headings are sufficiently broad as to allow inclusion of variations that deprescribing trials might include |
| 2 | Yes and No Yes because it will ensure that whatever deprescribing trials are being conducted are done to a certain standard and are consistent. However there aren't a lot of studies on deprescribing and if it is too 'mandated' or too 'rigid' then that may discourage studies |
| 2 | Too early |
| 2 | No need. |
| 2 | The same issues that apply to other trials apply to deprescribing trials. The CONSORT list applies if the study design dictates it. |
| 2 | I think deprescribing trials can be adequately described withing current CONSORT guidelines |
| 2 | Deprescribing trials are not different from the other types of clinical trials |
| 2 | I am not sure it needs to be amended, I believe most things could be fit under current headings, but perhaps an additional guidance/explanatory document explaining how to apply CONSORT to de-prescribing may be beneficial for some researchers, |
| 2 | I have insufficient knowledge to answer the question |
| 2 | CONSORT is an accepted standard and de-prescribing studies should be held to the same standard in order for results to be accepted by the research community and ultimately impact clinical care. |
| 2 | My english is very bad. I don't anderstand this question. Sorry! |
| 2 | These trial are the same as any other but you take something away rather than give it. |
| 2 | I do not think that a randomised trial could be the better desing for a deprescribing trial |
| 2 | The current classification can fit necessary points |
| 2 | it covers the necessary topics for interventions. |
| 2 | The criteria for CONSORT are the same for any kind of intervention (e.g. surgical, rehabilitative, pharmacologic) so deprescribing would fit under that, but would need each author/research group to clarify how each heading on the checklist applies. |
| 2 | the current list is appropriate |
| 2 | There are not specific concerns of deprescribing trials that are not addressed in this rubric. |
| 2 | we can use the available framework to fit deprescribing trials. |
| 2 | Deprescribing research can be written clearly within the current CONSORT framework |
| 2 | not sure -- no experience |
| 2 | Again, we need to make sure that we follow existing other regulatory processes |
| 2 | Too early at this stage |
| 2 | there are already way too many checklists!! it would be better to match the type of trial (e.g. there is one for non-charm interventions, one for cluster RCTs, and then SQUIRE etc for non randomised trials). WE need more hybrid trials focussing on implementation of the evidence rather than efficacy trials |
| 2 | not relevant |

Key:

GREEN & ORANGE = enough checklists/CONSORT list is sufficient (n = 23/34 respondents who gave comments saying an amendment to the CONSORT list is not required)

ORANGE = gave a similar response to question 11. (n = 6/72)
